# Growth factor levels in leukocyte-poor platelet-rich plasma and correlations with donor age, gender, and platelets in the Japanese population

**DOI:** 10.1186/s40634-019-0175-7

**Published:** 2019-02-02

**Authors:** Yu Taniguchi, Tomokazu Yoshioka, Hisashi Sugaya, Masahiko Gosho, Katsuya Aoto, Akihiro Kanamori, Masashi Yamazaki

**Affiliations:** 1 Department of Orthopaedic Surgery, Faculty of Medicine, University of Tsukuba, 1-1-1 Tennodai, Tsukuba, Ibaraki 305-8575 Japan; 2 Musculoskeletal System, Faculty of Medicine, University of Tsukuba, 1-1-1 Tennodai, Tsukuba, Ibaraki 305-8575 Japan; 3 Department of Clinical Trial and Clinical Epidemiology, Faculty of Medicine, University of Tsukuba, 1-1-1 Tennodai, Tsukuba, Ibaraki 305-8575 Japan

**Keywords:** Platelet-rich plasma, Age, Gender, Platelet count, Growth factor

## Abstract

**Background:**

Clinical application of platelet-rich-plasma (PRP) has been accelerated to investigate early recovery from various musculoskeletal conditions. It involves the promotion of tissue damage repair through the action of multiple growth factors at physiological concentrations. The composition of PRP differs based on many factors, which may include age and gender. Therefore, we analyzed correlations between age, gender, and platelet counts in PRP with growth factors in Japanese subjects.

**Method:**

Peripheral blood was drawn from 39 healthy volunteers between 20 and 49 years of age (age, mean ± standard deviation = 33 ± 8.7 years; gender ratio, male:female = 19:20; BMI, mean ± standard deviation = 22 ± 4.0) and prepared through centrifugation (volume, 6 mL per sample). After being activated with CaCl_2_, the supernatant was stored. The mean platelet count in PRP was 41.4 ± 12.2 × 10^4^/μL. PRP concentration rate (i.e., PRP/peripheral platelet counts) was 1.8 ± 0.4 times. Growth factor levels (platelet-derived growth factor-BB, transforming growth factor-β1, vascular endothelial growth factor, epidermal growth factor, fibroblast growth factor, insulin-like growth factor-1, and hepatocyte growth factor) were measured using enzyme-linked immunosorbent assay (ELISA), and correlations with age, gender, and PRP platelet counts were statistically analyzed by calculating Spearman’s rank correlation coefficients (r).

**Results:**

Age was negatively correlated with platelet-derived growth factor-BB and insulin-like growth factor-1 (*r* = − 0.32, − 0.39), and gender had no influence on growth factors. Platelet counts in PRP positively correlated with platelet-derived growth factor-BB, transforming growth factor-β1, epidermal growth factor, and hepatocyte growth factor (*r* = 0.39, 0.75, 0.71, and 0.48, respectively).

**Conclusions:**

This clinical study shows a significant variation of PRP among individual patients and that this variation is influenced by the age and the platelet counts of the subjects. Our data demonstrate that patient characteristics account for the differences in PRP physiological activity.

## Background

Platelet-rich-plasma (PRP) is defined as “the plasma fraction rich in platelets obtained from centrifuged whole blood” (Marx, 2001; Marx, 2004). Platelets contain different growth factors (GFs), a group of biologically active polypeptides that can stimulate cellular division, growth, and differentiation. In PRP therapy, various GFs in platelet-rich α-granules and GFs in the plasma as well as adhesion factors/glycoproteins are believed to act on tissue damage; moreover, this therapy is convenient and safe for the application of “autologous blood-derived products obtained through blood drawing” (Foster et al., 2009; Tayapongsak et al., 1994). In the past, PRP therapy has been conventionally used for bone formation and wound healing in oral surgery or plastic surgery. PRP therapy was first introduced by Tayapongsak et al. (1994) for mandibular reconstruction, and Whitman et al. (1997) started using it in dental/oral surgery in 3 years later. In the past decade, PRP therapy has been clinically applied in orthopedics to promote early recovery for various musculoskeletal conditions and then has expanded to include a wide variety of pathological conditions such as muscle strain, rotator cuff repair, knee osteoarthritis (OA), and tendinopathy (Fitzpatrick et al., 2017; Hamid et al., 2014; Reurink et al., 2014; Sanchez et al., 2012; Taniguchi et al., 2018; Zhang et al., 2016). However, a systematic review (Moraes et al., 2013), which evaluated PRP therapy for the treatment of musculoskeletal soft tissue injuries such as ligament, muscle and tendon tears, and tendinopathies and individual clinical conditions, concluded that evidence is insufficient to support the use of PRP for the treatment of musculoskeletal soft tissue injuries.

There are two major factors involved in the different clinical outcomes of PRP administration for various musculoskeletal conditions. First, devices from different manufacturers used to prepare PRP result in different quality. PRP with variations—including in platelet count, white blood cell (WBC) count, and some GFs—caused by various preparation protocols (the number of centrifugation steps, revolutions per minute, and the presence or absence of activation) has been reported (Castillo et al., 2011; Chahla et al., 2017; Kobayashi et al., 2016; Kushida et al., 2014; Oh et al., 2015). In order to determine the efficacy of PRP treatment, it is necessary to evaluate the composition of PRP prepared using various protocols. DeLong et al. (2012) and Mishra et al. (2012) proposed a mechanism to classify PRP. However, the devices and protocols used for PRP preparation are not the only factors contributing to wide disparities in biological activity; peripheral blood from an individual patient is characterized by a large inherent variability in the concentrations of platelets and GFs.

Recently, PRP has been used for various musculoskeletal conditions, and the age and gender of treated patients vary. Moreover, the age and gender affect biology and may also be important variables that influence the composition of PRP. However, only a few studies reported on correlations between GFs, age, and sex in PRP (Dragoo et al., 2012; Evanson et al., 2014; Weibrich et al., 2002; Xiong et al., 2017), and there is no report on the Japanese population in particular.

In this study, platelet and plasma-derived GF levels contained in leukocyte-poor PRP (LP-PRP) from Japanese subjects were exhaustively analyzed to provide basic data supporting the use of PRP therapy in the Japanese population. Specifically, we analyzed the influence of age, gender, and platelet counts in PRP on its quality.

## Methods

This study was reviewed and approved by the official ethical review board at our hospital. Prior to drawing blood, informed consent was obtained from each donor.

### Subjects and sample collection

Blood samples were collected from healthy individuals who donated blood during routine blood drives in the hospital. All identifiable subject information was omitted by the study’s personnel. The volunteers eligible for inclusion were male and female individuals from 20 to 49 years old who were not taking medication. Blood donors were provided with an information sheet prior to donating blood, which explained the purpose of the study and allowed them to decline to participate without affecting their ability to donate blood.

### PRP preparation

A 21 gauge butterfly needle was used to draw 36 mL of peripheral blood from the cubital vein of the subjects, avoiding hemolysis. Peripheral blood was collected into four 9-mL Spitz tubes containing 3.8% citric acid, centrifuged at 580×*g* for 8 minutes at room temperature (PRGF-Endoret® IV System; BTI Biotechnology Institute, Vitoria, Spain), and then separated into the erythrocyte layer, the buffy coat layer, and the plasma layer per Spitz tube. In the separated plasma layer, a safety area was set in the upper area of the buffy coat, avoiding aspiration of the buffy coat, and the upper half and lower half of the plasma layer were defined as platelet-poor-plasma (PPP) and platelet-rich-plasma (PRP), respectively. A dedicated aspirator in the PRGF®-Endoret® IV System (BTI Biotechnology Institute, Vitoria, Spain) was used for aspiration, and about 2 mL of PRP was collected per Spitz tube; thus, in total, about 8 mL of PRP was collected from the four tubes. The PRP was incubated at 37 °C for 1 hour after the addition of 5% calcium chloride (BTI Biotechnology Institute, Vitoria, Spain), and centrifuged at 1000×*g* for 20 min at 4 °C. The supernatant was then aspirated and stored at −80 °C.

### Hematological analysis

The WBC, red blood cell (RBC), and platelet counts in the whole-blood samples, PPP, and PRP were determined by using an automated cell count analyzer (Sysmex KX-21 N, Sysmex Corp., Kobe, Japan).

### GF quantification

The cryopreserved PRP was thawed at room temperature for use. Seven GFs were analyzed by using enzyme-linked immunosorbent assay (ELISA) kits specific for each GF (R&D Systems, Minneapolis, MN, USA). Platelet-derived growth factor (PDGF)-BB, transforming growth factor (TGF)-β1, vascular endothelial growth factor (VEGF), epidermal growth factor (EGF), fibroblast growth factor (FGF), insulin-like growth factor (IGF)-1, and hepatocyte growth factor (HGF) were measured according to the manufacturer’s recommendations. All standards and samples were analyzed in duplicate.

### Statistical analysis

No formal sample size justification was performed for this study, as it was an exploratory study. Kruskal-Wallis test was used for three-group comparison with post-hoc Fisher’s least significant difference (LSD) analysis. Wilcoxon’s rank sum test was performed for two-group comparison. The correlation of GFs with age, gender, and platelet counts in PRP was analyzed using the Spearman’s correlation coefficient. A statistical significance level of *P* < 0.05 was set in all tests. All data analyses were performed with JMP 10.0.2 (SAS Institute, Tokyo, Japan).

## Results

### Subject characteristics

Thirty-nine patients were enrolled in this study, including 19 females (48.7%) and 20 males (51.3%). Participants were classified into the following three age groups: 20–29, 30–39, and 40–49 years of age. Thirteen patients were 20 to 29 years (20s group), 13 patients were 30 to 39 years (30s group), and 13 patients were 40 to 49 years (40s group). The mean age (±standard deviation; range) was 33.9 (±8.7; 21–48 years), and the mean BMI was 21.4 (±4.0; 17.0–32.8 kg/m^2^).

### Hematological analysis in PRP and peripheral blood

The findings of the hematological analysis are presented in Table 1. The average platelet count in the PRP (41.4 ± 12.1 × 10^4^ /μL) was significantly higher than that in peripheral blood (23.4 ± 4.4 × 10^4^ /μL). The platelet count in PRP was 1.8-fold higher than that in peripheral blood. Based on the platelet count and the presence of WBC and neutrophils, the PRP preparation was classified as P2-x-Bβ according to the Platelets, Activation, and White cells classification system (DeLong et al., 2012).

**Table 1:** Hematological variables of each blood component

Whole blood	WBC (×10^2^ /μL)	57.8 ± 17.6
	RBC (×10^5^ /μL)	47.5 ± 4.8
	PLT (× 10^4^ /μL)	23.4 ± 4.4
PRP	WBC (×10^2^ /μL)	0.3 ± 0.8
	RBC (×10^5^ /μL)	0.2 ± 0.1
	PLT (×10^4^ /μL)	41.4 ± 12.2
PRP/Whole blood	ratio	1.8

Values in the table are shown as the average ± standard deviation

*WBC* white blood cells, *RBC* red blood cells, *PLT* platelet, *PRP* platelet-rich plasma

### Relationship between GF concentration and age, sex, and number of platelets in PRP

**Fig. 1:**
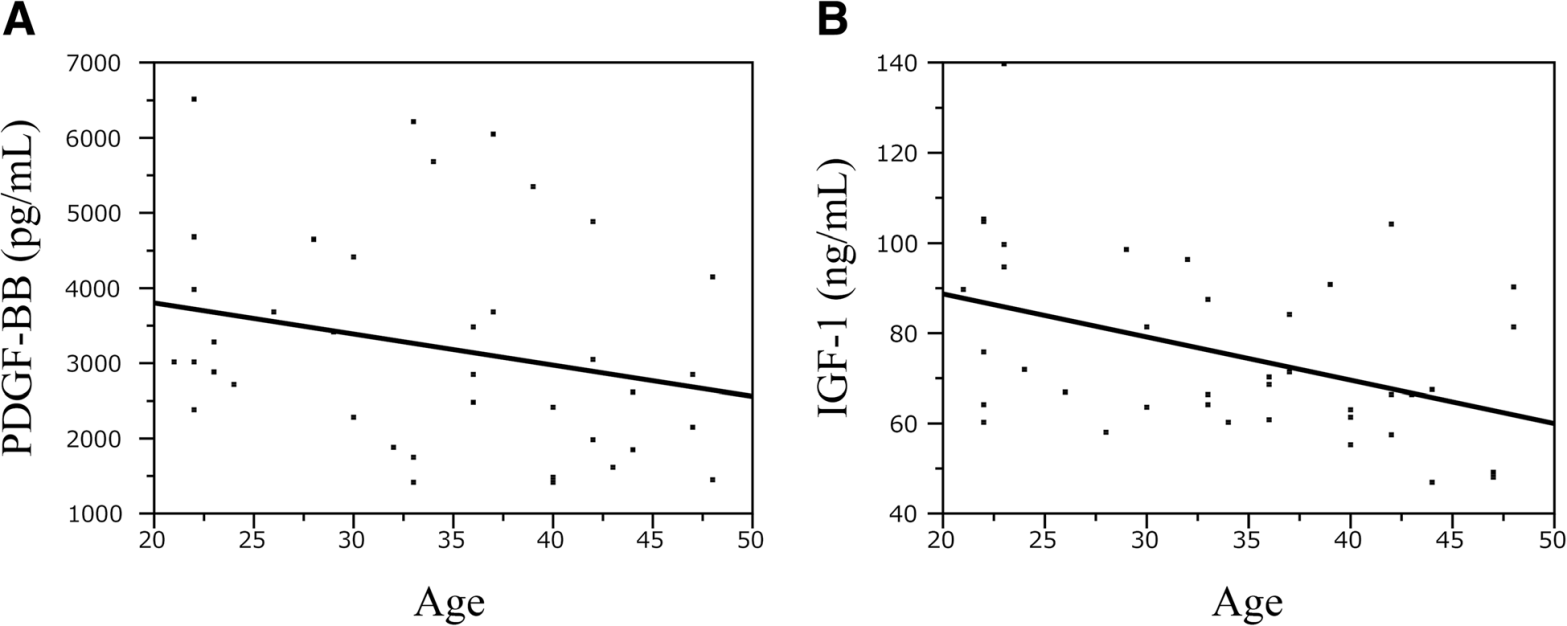
Correlation between the age of volunteers and concentrations of PDGF-BB and IGF-1 in PRP samples. PDGF-BB and IGF-1 levels significantly correlated with age (Spearman **a**: PDGF-BB r=-0.32, *p*<0.05, **b**: r=0.39, *p*<0.05)

The results of the immunoassays for the seven GFs and the platelet counts of whole blood and PRP are summarized in Table 2. In the age-group comparison (20s, 30s, and 40s), a significant difference was observed in PDGF-BB, EGF, IGF-1, and platelet counts in the whole blood and PRP. A negative correlation between age and levels of PDGF-BB and IGF-1 was detected with the Spearman correlation test (*p* = 0.049 and 0.014, respectively). The scatter diagram graphs of PDGF-BB and IGF-1 showing a negative correlation with age are shown in Fig. 1.

**Table 2:** Statistics including correlation with age for 10 analytes

Variable	20’s *N* = 13	30’s *N* = 13	40’s *N* = 13	P by Kruskal-Wallis test	*P* value for pairwise comparison*			Spearman’s correlation with age	*P*
					20’s vs 30 ‘s	20’s vs 40 ‘s	30’s vs 40 ‘s		
PDGF-BB (ng/mL)	3.3 [2.4, 6.5]	3.5 [1.4, 6.2]	2.1 [1.4, 4.9]	0.030	0.72	0.013	0.094	−0.32	0.049
TGF-β1 (ng/mL)	15.4 [11.0, 24.7]	17.1 [11.4, 31.9]	13.1 [11.2, 21.1]	0.12	–	–	–	−0.17	0.295
VEGF (pg/mL)	104 [21, 534]	290 [65, 591]	196 [41, 725]	0.20	–	–	–	0.19	0.325
EGF (pg/mL)	722 [489, 923]	861 [489, 1736]	612 [226, 938]	0.029	0.10	0.18	0.026	−0.19	0.255
FGF (pg/mL)	11 [5, 15]	12 [4, 40]	10 [4, 19]	0.33	–	–	–	−0.20	0.211
HGF (pg/mL)	431 [285, 596]	492 [357, 733]	451 [338, 560]	0.11	–	–	–	0.16	0.341
IGF-1 (ng/mL)	90 [58, 139]	70 [60, 96]	63 [47, 104]	0.027	0.18	0.024	0.10	−0.39	0.014
PLT (×10^4^/μL)	23 [16, 26]	26 [20, 34]	21 [18, 32]	0.004	0.003	0.94	0.024	0.03	0.87
PLT (PRP) (×10^4^/μL)	39 [28, 56]	47 [32, 82]	34 [23, 49]	0.004	0.077	0.16	0.001	−0.23	0.151

Values in the table are shown as median [minimum, maximum]

*PDGF-BB* platelet-derived growth factor-BB, *TGF-β1* transforming growth factors-β1, *VEGF* vascular endothelial growth factor, *EGF* epidermal growth factor, *FGF* fibroblast growth factor, *HGF* hepatocyte growth factor, *IGF-1* insulin-like growth factor-1

*Fisher’s LSD for multiplicity adjustment

**Fig. 2:**
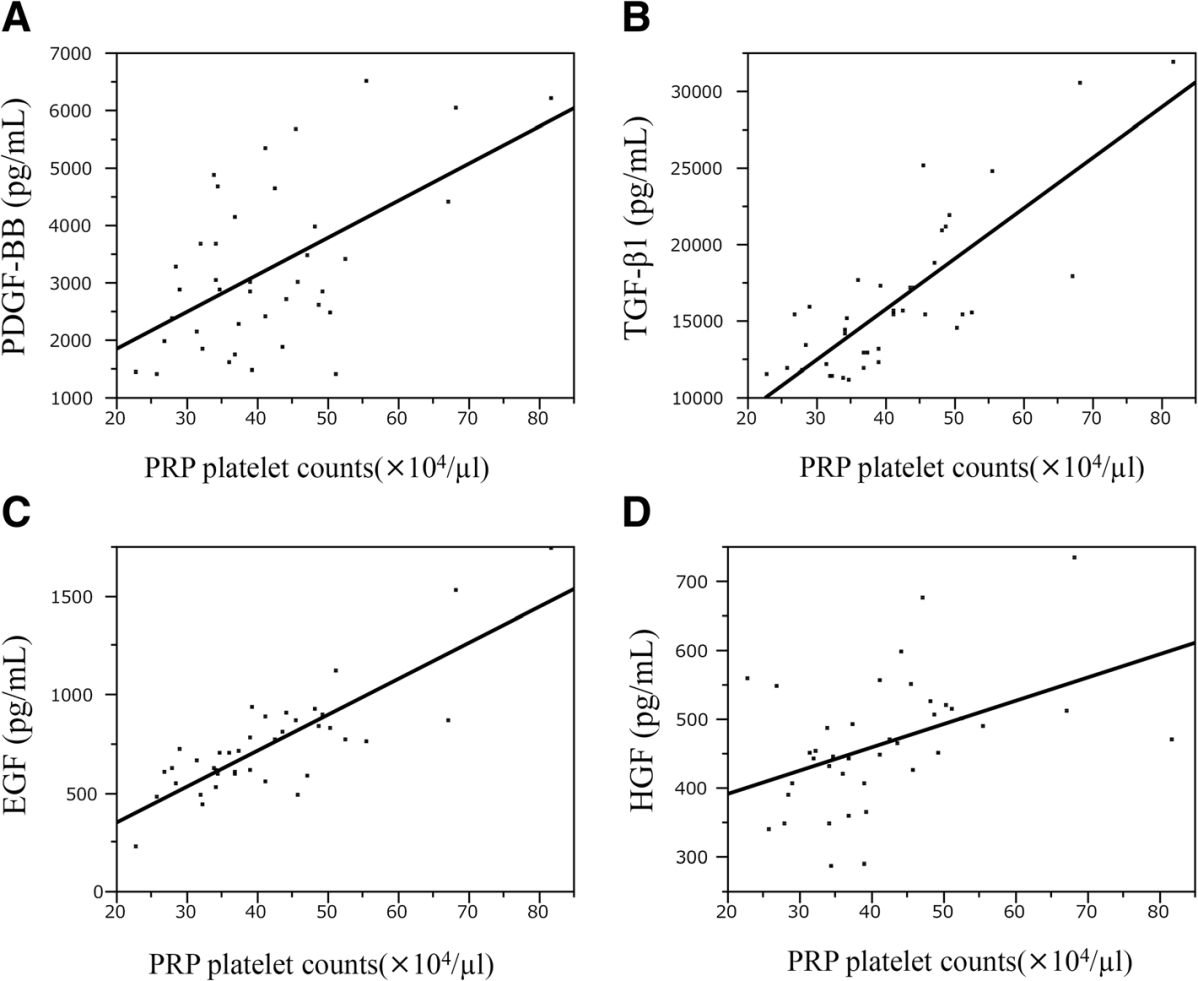
(**a**: PDGF-BB r=0. 39, p<0.05, **b**: TGF-β1 r=0. 75, p<0.001, **c**: EGF r=0.71, *p*<0.001, **d**: HGF r=0.48, *p*<0.05)

In this study, no significant difference in GF levels between male and female subjects was observed (Table 3). A correlation was observed between platelet counts in PRP and levels of PDGF-BB, TGF-β1, EGF, and HGF (Table 4). Scatter diagram graphs showing these correlations are shown in Fig. 2.

**Table 3:** Various factors’ concentrations as a function of gender

Variable	Male	Female	P by Wilcoxon’s rank sum test
	*N* = 20	*N* = 19	
PDGF-BB (ng/ml)	3.5 [1.4, 6.5]	2.9 [1.5, 5.3]	0.31
TGF-β1(ng/ml)	15.6 [11.3, 31.9]	15.2 [11.1, 18.7]	0.20
VEGF (pg/ml)	240 [41, 591]	140 [21, 725]	0.47
IGF-1 (ng/ml)	68 [47, 105]	70 [48, 139]	0.40
EGF (pg/ml)	765 [226, 1736]	697 [489, 938]	0.69
FGF (pg/ml)	11 [4, 40]	10 [4, 20]	0.35
HGF (pg/ml)	479 [338, 733]	443 [285, 675]	0.074
PLT (×10^4^ /μL)	23 [16, 34]	24 [18, 31]	0.87
PLT (PRP) (×10^4^ /μL)	43 [23, 82]	37 [27, 53]	0.37

Values in the table are shown as median [minimum, maximum]

**Table 4:** Spearman’s correlation coefficients for platelet counts in platelet rich plasma and growth factor levels

Growth factors	Correlation of platelet counts in PRP	*p*
PDGF-BB	0.39	0.015
TGF-β1	0.75	< 0.001
VEGF	0.08	0.69
EGF	0.71	< 0.001
FGF	0.17	0.296
HGF	0.48	0.002
IGF-1	−0.08	0.63

## Discussion

The physiological effect of PRP therapy on tissue damage is exerted through the integrated action of various GFs in platelet α-granules and GFs and glycoproteins (adhesion factors) contained in the plasma, while maintaining an equilibrium in vivo (Alsousou et al., 2009; Foster et al., 2009). GFs play a vital role in the recovery from injury of cartilage, ligaments, and tendons (Fortier et al., 2011; Molloy et al., 2003; Oliva et al., 2012). In this study, seven GFs in LP-PRP used in clinical practice were measured to analyze their correlations with age, gender, and platelet counts in PRP. This study provided the following information. First, a negative correlation was observed between age and the levels of PDGF-BB and IGF-1, two of the measured GFs. No correlation was detected between age and the other five GFs. Secondly, no correlation was detected between gender and levels of GFs in PRP. Finally, a positive correlation was observed between four of the seven GFs (PDGF-BB, TGF-β1, EGF, and HGF) and platelet counts in PRP. Additionally, GF levels were higher in subjects with greater platelet counts.

Some studies analyzed the relationship between age and gender and GFs contained in PRP. Evanson et al. (2014) reported that levels of PDGF-BB, TGF-β1, IGF-1, and EGF in the LP-PRP were statistically higher in subjects younger than 25 years old compared to those in subjects aged 26 years old or older. Additionally, some other studies reported a weak negative correlation between age and levels of PDGF-BB and IGF-1, but these studies did not describe the details of purified PRP (Cho et al., 2011; Dragoo et al., 2012; Weibrich et al., 2002). However, Anitua et al. reported that IGF-I levels were significantly reduced and HGF levels were increased in elderly individuals compared with the young group (Anitua et al., 2013). The results of this study indicate a correlation between age and levels of PDGF-BB and IGF-1 in the LP-PRP, consistent with previous studies. Moreover, the correlations between age and PDGF-BB (*r* = − 0.32) or IGF-1 (*r* = − 0.39) were weakly negative correlations, and no correlation was observed with the other five types of GFs (i.e., TGF-β1, VEGF, FGF, EGF, and HGF). PDGF is a potent mitogen and chemotactic factor for cells of mesenchymal origin, on which it demonstrates a major proliferative effect as well as inhibition of differentiation (Husmann et al., 1996). Also, PDGF-BB is one of the three isoforms of PDGF; with regard to muscle precursor cells in vitro, PDGF-BB showed a significant promotion of proliferation and inhibition of differentiation (Jin et al., 1990). IGF-1 also plays an important role in muscle regeneration, and its action has been clarified. It promotes both proliferation and differentiation of myoblasts, induces myofiber hypertrophy, and protects from atrophy. IGF-1 also increases myoblast proliferation (Barton-Davis et al., 1999) and myofiber protein synthesis (Bark et al., 1998). In other words, since both PDGF-BB and IGF-1 have a function to proliferate muscle cells in muscle regeneration, the results of this study suggest that LP-PRP prepared from young patients may be more effective for various musculoskeletal conditions in the clinical setting. In addition, PDGF-BB and IGF-1 inhibit nuclear factor-κB (NF-κB) activity and apoptosis via suppression of IL-1β (Montaseri et al., 2011). Since NF-κB plays a crucial role in OA, leading to cartilage destruction and articular damage (Roman-Blas & Jimenez, 2006), when using PRP for the treatment of OA, age must also be taken into consideration.

No significant difference was observed between gender and GFs in PRP in this study. A few studies reported a correlation between gender and GFs (Evanson et al., 2014; Weibrich et al., 2002; Xiong et al., 2017), but these results were inconsistent. Hormonal differences drive many sex-related differences between men and women. In particular, menstruation in women has a significant impact on hormones; increases in inflammatory markers were observed under the influence of menopause in some studies (Pfeilschifter et al., 2002; Singh & Newman, 2011). Subjects in this study were healthy adults between 21 and 48 years of age, and although the menstrual cycle was not taken into account, the female subjects were not affected by menopause. There was a possibility that the difference in the target age group influenced the results of this study.

There are some reports on platelet counts in PRP and GF levels (Anitua et al., 2009; Eppley et al., 2004; Magalon et al., 2014; McCarrel & Fortier, 2009; Sanchez et al., 2007). Despite earlier reports in the literature, the platelet number in PRP is positively correlated with GF concentration, but it is reported that GFs are not enriched as much as platelets and that there is a difference in concentration rate depending on the type of GFs. Several GFs are stored not only in platelets but also in leukocytes (Boswell et al., 2012) and are affected by the number of WBC in the PRP (Sundman et al., 2011). In this study, we investigated PDGF-BB, TGF-β1, VEGF, EGF, FGF, and HGF as GFs contained in the α-granules of platelets and IGF-1 as a GF contained in plasma but not platelets. The GFs that correlated with the platelet count in LP-PRP were PDGF-BB, TGF-β1, EGF, and HGF; the GF that showed the strongest positive correlation among them was TGF-β1. TGF-β is a multifunctional cytokine, which plays an important role in regulating repair and remodeling following tissue injury (Husmann et al., 1996). Following trauma, TGF-β is expressed by regenerating muscles, suggesting that it may play a role in muscle regeneration (Bachl et al., 2009). However, TGF-β1 has the ability to inhibit myogenic differentiation, myoblast fusion, and the expression of various muscle-specific proteins (Florini et al., 1991; Liu et al., 2001). Moreover, since TGF-β1 contributes to increased collagen deposition, causing muscle fibrosis, experimental studies have been conducted to antagonize TGF in PRP to suppress myofibrosis (Li et al., 2016; Terada et al., 2013). On the other hand, for articular cartilage, TGF-β1 is considered as an anabolic factor and capable of activating the chondrogenic properties of reparative mesenchymal stem cells in vitro (Johnstone et al., 1998; Mackay et al., 1998). Also, TGF-β1 required for the normal development of a joint, and the lack of its signaling can cause a normal joint to develop into an osteoarthritic joint. When PRP is used for various musculoskeletal conditions, there are scenes where TGF-β1 is required and not necessary, and the optimum PRP varies depending on the environment in which it is used. A correlation was observed between platelet counts in PRP and VEGF levels in the above studies; however, although the above studies used leukocyte-rich PRP, and LP-PRP was used in this study, the average was only 300/μL. The lack of correlation with VEGF can be explained by the fact that only a few leukocytes are contained in PRP, as a positive correlation has been reported between GFs and leukocyte counts in PRP (Castillo et al., 2011; Magalon et al., 2014). Interestingly, a positive correlation was observed between platelet counts in PRP and platelet-derived HGF in this study, which has not been reported previously. HGF is an inhibitor of NF-κB, one of the main transcription factors that controls the inflammation process (Bendinelli et al., 2010). PRP contains various platelet-derived GFs, functioning in accordance with the environment of use and affecting the cells and tissues targeted by each GF. Treatment outcome may also be influenced by the combined effects of GFs in some cases (Engebretsen et al., 2010; Foster et al., 2009). Previously, PRP GF concentration was generally considered to be proportional to platelet concentration. However, the results of this study indicate that even for platelet-derived GFs, not all GF concentrations are necessarily proportional to platelet concentration. Thus, when implementing PRP treatment for tissues and organs targeted by these GFs, the GF concentration needs to be assessed separately from platelet concentration. In effect, the “more is better” theory does not appear to be valid for platelet concentration in PRP (Giusti et al., 2014). Rather, an appropriate type of PRP needs to be selected in accordance with the target tissue and environment of use.

While this study investigated inherent differences among individual patients in the PRP composition, host factors may also affect responses to PRP treatment. For example, the younger the age in this study, the higher the IGF-1 concentration in PRP, but the response of tissues to GFs varies based on host characteristics such as age and gender. Furthermore, subject age may affect the potential for regenerative responses. As previously mentioned, older, arthritic chondrocytes show a blunted response to IGF-1 (Loeser et al., 2014; Verschure et al., 1995). For future research, based on the results of this study, the impact of host factors on PRP treatment should also be considered.

This study has some limitations. First, the subject age groups were limited. As this study was designed to include subjects in their 20s, 30s, and 40s with athletes in mind, data obtained from this study do not provide information for the middle-aged population of 50 years of age or older. As PRP is used in injection therapy for osteoarthritis of the knee, data from the population of 50 years of age or older are required in the future. Secondly, the results of this study are mostly exploratory in nature because the sample size was insufficient to confirm the relationship between GFs and age and gender, respectively. Thus, the findings should be interpreted cautiously.

## Conclusion

In this study, GF concentrations contained in LP-PRP were analyzed. A negative correlation was observed between age and levels of PDGF-BB and IGF-1, while a positive correlation was detected between platelet counts in PRP and levels of PDGF-BB, TGF-β1, EGF, and HGF. Gender had no significant influence on GF levels. The results of this study are important basic data for the use of LP-PRP in clinical practice.

## Abbreviations

EGF: Epidermal growth factor
FGF: Fibroblast growth factor
HGF: Hepatocyte growth factor
IGF: Insulin-like growth factor
LP-PRP: Leukocyte-poor PRP
LSD: Least significant difference
NF-κB: Nuclear factor-κB
OA: Osteoarthritis
PDGF: Platelet-derived growth factor
PLT: Platelet
PPP: Platelet-poor plasma
PRP: Platelet-rich plasma
RBC: Red blood cells
TGF: Transforming growth factor
VEGF: Vascular endothelial growth factor
WBC: White blood cells
